# SIX3 and SIX6 interact with GEMININ via C-terminal regions

**DOI:** 10.1016/j.bbrep.2019.100695

**Published:** 2019-10-23

**Authors:** Diana C. Turcu, Johan R. Lillehaug, Hee-Chan Seo

**Affiliations:** Department of Biological Sciences, University of Bergen, Bergen, Norway

**Keywords:** SIX3, SIX6, GEMININ, Cell-cycle regulation, Bimolecular fluorescence complementation (BiFC), Surface plasmon resonance (SPR), Isothermal titration calorimetry (ITC), Protein interaction

## Abstract

The histoarchitecture and function of eye and forebrain depend on a well-controlled balance between cell proliferation and differentiation. For example, the binding of the cell cycle regulator GEMININ to CDT1, which is a part of the pre-replication complex, promotes cell differentiation. Homeodomain transcription factors SIX3 and SIX6 also interact with GEMININ of which SIX3-GEMININ interaction promotes cell proliferation, whereas the nature of SIX6-GEMININ interaction has not been studied to date. We investigated SIX3/SIX6 and GEMININ interactions using bimolecular fluorescence complementation, surface plasmon resonance and isothermal titration calorimetry. Interactions between SIX3/SIX6 and GEMININ were detected in mammalian cells in culture. The presence of the C-terminal regions of SIX3 and SIX6 proteins, but not their SIX domains or homeodomains as previously thought, were required for interaction with GEMININ. Interestingly, the disordered C- and N- terminal regions of GEMININ were involved in binding to SIX3/SIX6. The coiled-coil region of GEMININ, which is the known protein-binding domain and also interacts with CDT1, was not involved in GEMININ-SIX3/SIX6 interaction. Using SPR and ITC, SIX3 bound GEMININ with a micromolar affinity and the binding stoichiometry was 1:2 (SIX3 - GEMININ). The present study gives new insights into the binding properties of SIX proteins, especially the role of their variable and disordered C-terminal regions.

## Highlights

• C-terminal regions of SIX3/SIX6 bind GEMININ.
• GEMININ coiled-coil region is not involved in SIX3/SIX6 interaction.
• C- and N-terminal regions of GEMININ bind SIX3/SIX6.
• SIX3 binds GEMININ with a binding stoichiometry of 1:2.

## 1 Introduction

The homeodomain-containing transcription factors SIX3 and SIX6 play a crucial role in eye and forebrain development in vertebrates [[1], [2], [3]]. They influence the expression of various proteins through the direct binding to their regulatory DNA sequences as well as through the interaction with other proteins (co-factors) [4], for example, the DNA replication inhibitor GEMININ [5]. GEMININ itself inhibits cell cycle progression by binding to CDT1 (part of the pre-replication complex) [[6], [7], [8], [9]] and by controlling CDT1 levels [10]. Interestingly, both SIX3/SIX6 and CDT1 compete for the binding to GEMININ [5]. Thus, the competition between SIX3/SIX6 and CDT1 for GEMININ may be important for gene expression and biological cell fate. The present study focuses on SIX-GEMININ binding properties.

Mammals have 6 SIX proteins divided into 3 groups based on their primary protein structure and gene expression patterns: the SIX1/2, SIX3/6 and SIX4/5 groups [11]. All members of the SIX class have two highly conserved structural domains, a SIX domain (SD) and an adjacent homeodomain (HD). The genes belonging to the SIX3/6 group are mainly expressed in the neuronal tissues and, in case of SIX3 and SIX6, their homeodomains differ only in a single amino acid (98.3% sequence identities) and their SIX domains are also highly similar (sequence identities, 88.2%; sequence similarities, 98.4%). The helix-turn-helix shaped homeodomain features specific DNA binding sites, while the SIX domain, which contains 6 α helices, is involved in protein interactions [[12], [13], [14]]. The N-terminal region of SIX6, the upstream of the SIX domain, is very short (8 aa) and lacks the first 78 amino acids (including the Gly-rich region) present in SIX3. SIX3 and SIX6 are also different in their C-terminal regions, with the exception of the first 8 amino acids and the last 15 amino acids that are highly conserved. The function of these variable C-terminal regions is currently not known. However, in SIX6 the 15 aa adjacent to the C- terminus of the homeodomain was shown to render a DNA binding specificity that is not present in SIX2, another member of the SIX class [15].

The human GEMININ is 209 aa-long and the 3D-structure of its protein interaction domain, the coiled-coil domain (aa 91–160), has been determined [[16], [17], [18]]. GEMININ homo-dimerises through the coiled-coil domain and exists as a tetramer under low salt condition (200 mM salt or lower).

The binding of GEMININ on CDT1 inhibits MCM (mini chromosome maintenance) loading [6,8] and affects histone acetylation [7] and deacetylation [9]. GEMININ also promotes the accumulation of CDT1 during mitosis. In metaphase, GEMININ is degraded via APC (anaphase-promoting complex), leading to the activation of CDT1 in early G1 for pre-RC (pre-replication complexes) formation [10]. The CDT1-GEMININ complex can switch between a licensing permissive heterotrimer and a licensing inhibitory heterohexamer state depending on the concentration of GEMININ, and the coiled-coil domain of GEMININ is involved in the CDT1 interaction [16,19].

Previous protein interaction studies (yeast two-hybrid screen and pull-down assay) have shown that GEMININ could bind both SIX3 and SIX6 [5], and EMSA (electromobility shift assay) revealed that SIX3 could simultaneously bind to DNA and to GEMININ. The GEMININ-SIX3 interaction was further studied by knock-out as well as overexpression studies, revealing SIX3 and GEMININ act antagonistically to regulate the balance between proliferation and differentiation of cells. Recent studies showed that GEMININ binds the homeodomains of HOX proteins through its homeodomain binding region (HBR, 171–190 aa) [20,21]. However, the homeodomain of the SIX3 protein did not bind to the HBR of GEMININ [21], indicating that GEMININ may bind a different region of SIX3, for instance, the conserved protein-protein interacting SIX domain. To date, no data on the SIX6-GEMININ interaction is available.

The lack of *SIX3/SIX6* genes and/or their improper regulations lead to death, inborn defects, or abnormalities (e.g., holoprosencephaly), and cancers [[22], [23], [24], [25]]. The importance of these genes in animal development was studied by multiple knock-out [22], gain- and loss-of-function analyses [24,25]. However, still very little is known about the mechanism by which SIX3/SIX6 proteins interact with other proteins, and especially with GEMININ.

In the present study, we show that the bindings of SIX3/SIX6 protein to GEMININ requires the presence of their variable C- terminal regions and that GEMININ itself does not use the well-known coiled-coil region, but its C- and N- terminal regions. We also provided, albeit limited, thermodynamical parameters, stoichiometry and kinetics of such interaction, which in turn would lead to new insight in the nature of interaction involving these important transcription factors.

## 2 Materials and methods

### 2.1 Molecular cloning

The full-length coding sequences of the human *SIX3*, *SIX6* and *GEMININ* were chemically synthesised and cloned into either pMA (*SIX3* and *SIX6*) or pMK (*GEMININ*) (GeneArt). The *SIX3* sequences encoding a SIX3 protein without the N-terminal homopolymeric region (aa 1–78, GenBank AF049339) were hereto named full-length on a par with the full-length SIX6 protein, which does not contain this homopolymeric region. All deletion constructs were generated by site-directed mutagenesis (SDM) and confirmed by sequencing. The primer pairs used were: Sequences encoding SIX3/SIX6 SIX domains (including 8 extra conserved amino acids at the N-terminus), 5′ ACC ATT TGG GAT **T**G**A** GAA CAG AAA ACC and 5′ GGT TTT CTG TTC **T**C**A** ATC CCA AAT GGT (for introduction of a stop codon, shown in bold here and elsewhere); SIX3/SIX6 homeodomains, 5′ CCA TGA GCT CCC ATG GAT GGT GAA CAG AAA ACC CAT TGC and 5′ GCA ATG GGT TTT CTG TTC ACC ATC CAT GGG AGC TCA TGG (first for making the homeodomain and C-term), and 5′ CGT GCA GCA GCA **TG**A AAA AAT CGT CTG and 5′ CAG ACG ATT TTT T**CA** TGC TGC TGC ACG (for subsequent introduction of a stop codon); SIX3/SIX6 C-terminal regions, 5′ CCA TGA GCT CCC ATG GAT GCA AAA AAT CGT CTG CAG and 5′ CTG CAG ACG ATT TTT TGC ATC CAT GGG AGC TCA TGG. GEMININ coiled-coil region (aa 82–160), 5′ CCA TGA GCT CCC ATG GAT ACC CAA GAA TCC TTT GAT CTG ATG and 5′ CAT CAG ATC AAA GGA TTC TTG GGT ATC CAT GGG AGC TCA TGG (for deletion of the N-terminus), and 5′ CGT CTG AAT GGT **T**AA CCG CTG GAT and 5′ ATC CAG CGG TT**A** ACC ATT CAG ACG (for subsequent introduction of a stop codon); GEMININ N-terminal region (aa 1–81), 5′ CTG GGT GGT GTT **TGA** CAA GAA TCC TTT GAT C and 5′ GAT CAA AGG ATT CTT G**TC A**AA CAC CAC CCA G (for introduction of a stop codon); GEMININ C-terminal region (aa 161–209), 5′ CCA TGA GCT CCC ATG GAT GAA CCG CTG GAT AAT TTT GAA AGC and 5’ GCT TTC AAA ATT ATC CAG CGG TTC ATC CAT GGG AGC TCA TGG.

All three genes and their deletion constructs were cloned into the NcoI and BamHI sites of pETMBP_1 (gift from G. Stier, EMBL), so that expressed proteins would have MBP and 6x His tags and a TEV cleavage site in their N- termini. The EcoRI and BglII sites were utilised in cloning of the constructs used in bimolecular fluorescence complementation (BiFC).

### 2.2 Expression and purification of SIX3/SIX6 and GEMININ proteins

The plasmids encoding proteins of interest were transformed into *Escherichia coli* strain BL21Star™ (DE3) (Invitrogen) and were grown in lysogeny broth (LB) containing kanamycin (50 μg/ml). Cells were first grown at 37 °C for 3 h, and induced when A_600_ reached 0.8 with 0.5 mM isopropyl 1-thio-*β*-d-galactopyranoside (IPTG). Cells were further grown at 18 °C overnight and were harvested by centrifugation at 9000×*g*. The cell pellets were resuspended in ice-cold lysis buffer (25 ml for 500 ml culture). The composition of lysis buffer was: 1x PBS, 300 mM NaCl, 2 mM DTT, 0.5% NP 40, protease inhibitor (Sigma, 1 tablet of Complete EDTA-free per 100 ml lysis buffer).

Recombinant proteins were purified using metal affinity (Ni-NTA resin) on HisTrap HP and size-exclusion HiLoad 16/60 Superdex 75/200 columns from GE Healthcare. Peak fractions were collected and verified by SDS-PAGE. The final buffer composition was: 1xPBS, 350 mM NaCl, 3.5 mM tris(2-carboxyethyl)phosphine (TCEP), protease inhibitor.

Since the SIX3/SIX6 proteins were unstable without a tag, the MBP tag was not removed upon purification and MBP-fusion proteins were used in SPR and ITC measurements. For GEMININ, the MBP tag was removed by TEV digestion before the final purification.

Purified proteins were concentrated using an Amicon Ultra 30 device and aliquots were stored for 1–2 days at 4 °C. Protein concentration was estimated at A_280_ using a microplate spectrophotometer (BioTek) and the concentration was calculated using the theoretical extinction coefficients provided by ProtParam.

### 2.3 Surface plasmon resonance (SPR)

All sensorgrams were recorded on Biacore T200 (GE Healthcare) using CM5 sensor chips (GE Healthcare). Prior to analysis, proteins were centrifuged for 30 min  at 16 000×*g* at 4 °C and the concentration was measured again. The interaction between the immobilised ligand (GEMININ) and the mobile-phased analyte (MBP-fused full-length SIX3 or SIX domain of SIX3) was analysed. MBP alone was also used as analyte to serve as a control. The ligand was immobilised by standard amine coupling on one of the flow cell on the CM5 dextran-coated sensor chip, while the other flow cell was used as a reference after 1-ethyl-3-(3-dimethylaminopropyl)carbodiimide (EDC), N-hydroxysuccinimide (NHS), ethanolamine activation followed by ethanolamine blocking.

Non-specifically immobilised ligand was removed from the CM5 chips by washing for several hours at 60 μl/min with running buffer ((PBS-TCEP (3.5 mM TCEP in 1xPBS), 350 mM NaCl) prior to SPR analysis. All experiments were performed at 25 °C in running buffer at a flow rate of 30 μl/min. Different concentrations of analyte were injected during the association phase for 240 s and the chip surface was exposed to running buffer for 600 s to monitor the dissociation phase. The chip surface was regenerated by the injection of 30 μl/min of regeneration buffer (10 mM glycine, pH 2.2).

The SPR data were analysed with the Biacore T200 Evaluation Software. Double referencing (subtracting a blank run with running buffer after subtracting the reference cell) was applied to all curves. The apparent equilibrium dissociation constant was estimated from plotting the response at the end of the injection against the analyte concentration using a steady-state fit.

### 2.4 Isothermal titration calorimetry (ITC)

ITC was performed using a Nano ITC LV (TA Instruments) with degassed buffers at 25 °C. To eliminate possible heat changes due to buffer effects, protein samples were purified in the reaction buffer containing (PBS-TCEP, 300 mM NaCl). Proteins were centrifuged for 30 min  at 16 000×*g* at 4 °C and the concentration was estimated again. GEMININ was used in the titration syringe, and MBPSIX3 was used in the sample cell. ITC data collection consisted of 21 titrant injections (2 μl each) into the sample cell at time intervals of 180 s. Control titrations (GEMININ and MBP alone) were subtracted from the raw data. Raw ITC data were integrated and analysed using built-in binding models of the NanoAnalyzer software from TA Instruments. For quality control, after the experiment was completed, the proteins were removed from the cell and checked on size exclusion chromatography using HiLoad 16/60 Superdex 200 column.

### 2.5 Bimolecular fluorescence complementation (BiFC)

The gene sequences of *SIX3*, *SIX6* and *GEMININ* were used in cloning of pBiFC-VC155 (the C-terminal half of Venus protein, HA tag) and pBiFC-VN155 (I152L) (the N-terminal half of Venus protein, Myc tag) expression vectors [26] (Addgene). Both N and C combinations of SIX3 or SIX6 and GEMININ were tested. As no differences were observed when using N-terminal versus C-terminal construct, the main experiments were performed with SIX3/SIX6 constructs in the pBiFC-VC155 expression vectors and GEMININ constructs in the pBiFC-VN155 (I152L) vectors. The COS-1 cells (Sigma-Aldrich nr. 88031701, SV40 transformed) were cultured at 37 °C in a humidified 5% CO_2_ incubator in DMEM supplemented with 10% fetal bovine serum and antibiotics (100 IU/ml penicillin and 100 μg/ml streptomycin). For transfections, subconfluent 100-mm plates of COS-1 were split 1:6 into 6-well plates containing two cover-slips per well. 500 ng pBiFC-VC155 and 500 ng pBiFC-VN155 (I152L) constructs were used for transfection the following day using TurboFect (Fermentas) according to the instructions manual and were incubated at 37 °C in a CO_2_ incubator. The transfection efficiencies of the constructs encoding homeodomain were low, whereas other constructs gave comparably good transfections.

At 24 h post-transfection, the 12 mm coverslips covered with cells were washed in PBS (phosphate buffered saline). Cells were fixed in 3.7% paraformaldehyde in PBS for 8 min, washed three times in PBS and permeabilised with 0.25% Triton-X100 in PBS for 10 min at room temperature. After washing twice in PBS, the cells were incubated with blocking solution (5% goat serum in PBST (1% Triton in PBS)) for 1.5 h. Primary antibodies anti-Myc (rabbit polyclonal, Abcam, ab9106), anti-HA (mouse monoclonal, Covance, MMS-101P) were used at 1:500 dilution for 2 h at room temp. Cells were washed four times for 5 min in PBS at room temp. Incubation with a second antibody (1:200 dilution) was carried out in the dark for 1 h at room temperature. Secondary antibodies used were goat Alexa 594 (Life Technologies) either anti-mouse (A11005) or anti-rabbit (A11012). Afterwards, cells were extensively washed in PBS and coverslips were mounted with the Prolong Gold antifade reagent and incubated in the dark for 24 h. Images were obtained using Leica TCS SP5 confocal microscope. Approximately 100 individual cells from three biological replicates were subjected to image analysis as described previously [27]. Obtained mean pixel intensities from BiFC were plotted against the immunofluorescence signal.

## 3 Results

### 3.1 Full-length SIX3, but not the SIX domain alone, binds GEMININ in SPR studies

The interactions between SIX3 and GEMININ were investigated by surface plasmon resonance (SPR). We immobilised GEMININ as a ligand on CM5 chip surface and analysed association and dissociation kinetics of MBP alone (Fig. S4), MBP-fused full-length SIX3 (Fig. 1) and MBP-fused SIX3SD (Fig. 1) as analytes at various concentrations in multiple cycle experiments. The experiments were done at high salt (437 mM NaCl) concentrations, at which SIX3 exists in a monomeric form. The apparent dissociation equilibrium constant, K_D_, was estimated by plotting the response at the end of the injection against the analyte concentration using a simple steady-state fit. The fitted curves for SIX3 (Fig. 1B) showed an affinity in the micromolar range (apparent K_D_ = 3.19 μM). The SIX3SD showed approximately a 6-fold reduced ability to bind GEMININ (apparent K_D_ = 19.5 μM) compared with the full-length SIX3 protein (Fig. 1D). The SIX3SD-GEMININ binding curves also showed a markedly gentler association and dissociation phase (Fig. 1C). The binding of MBP alone to GEMININ (Fig. S4A) was 12 fold less than that of MBPSIX3-GEMININ (apparent K_D_ = 35.3 μM).

**Fig. 1:**
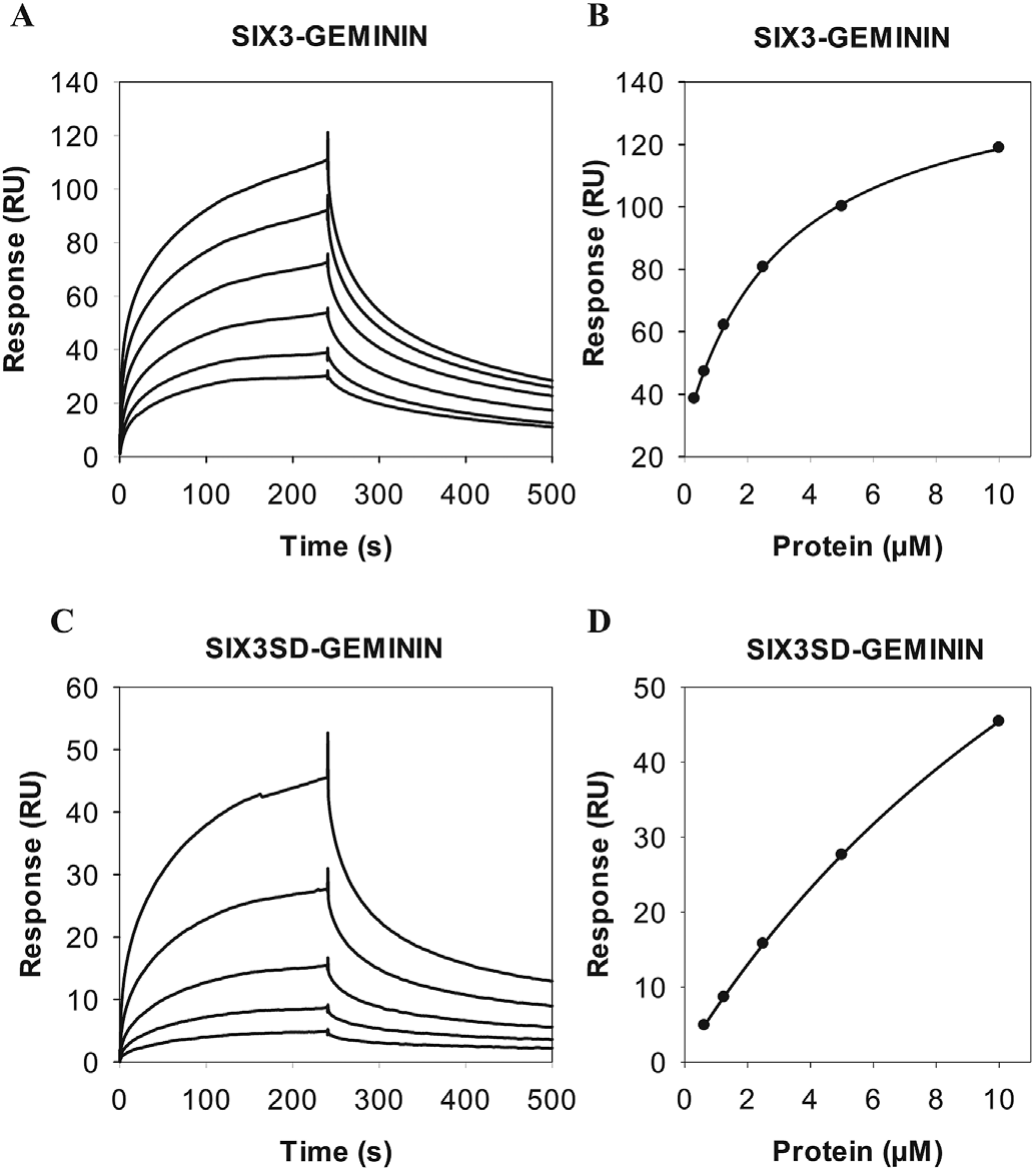
**SPR analysis of SIX3 and SIX3SD with GEMININ**. 1370 RU GEMININ were immobilised on the chip. A) SIX3-GEMININ sensorgram. MBPSIX3, at varying concentrations of 0.31 μM, 0.62 μM, 1.25 μM, 2.5 μM, 5 μM, 10 μM was injected into the GEMININ-containing flow cell and the reference flow cell for 240 s. B) SIX3-GEMININ binding affinity curve: K_D_: 3.19 μM, R_max_: 120, Chi^2^ (RU^2^): 0.34. C) SIX3SD (SIX domain of SIX3)-GEMININ sensorgram. MBPSIX3SD, at varying concentrations of 0.62 μM, 1.25 μM, 2.5 μM, 5 μM, 10 μM, was injected into the GEMININ-containing flow cell and the reference flow cell for 240 s. D) SIX3SD-GEMININ binding affinity curve: K_D_: 19.6 μM, R_max_: 132, Chi^2^ (RU^2^): 0.003.

### 3.2 SIX3 binds GEMININ in a 1:2 ratio and the coiled-coil region of GEMININ is not involved

We used isothermal titration calorimetry (ITC) to study the stoichiometry and the thermodynamics of SIX3 binding to GEMININ. The binding experiments were performed at high salt (437 mM NaCl) concentrations as in the SPR experiments, with 200 μM GEMININ in the titration syringe and 10 μM MBPSIX3 in the cell. In this high salt buffer, SIX3 exists as a monomer, whereas GEMININ exists as a dimer. Under these experimental conditions, the monomeric MBPSIX3 bound to the dimeric GEMININ (Fig. 2A and B) whereas MBP alone with GEMININ had no such binding (Figs. S4C and D). The fitted binding isotherm (for independent binding sites) reveals an n value of 0.55, indicating a stoichiometry of 1 (SIX3): 2 (GEMININ) for this interaction (Fig. 2B). The values for the thermodynamic parameters (ΔH = -317 kJ/mol and ΔS = -957 J/molK) and equilibrium dissociation constant (K_D_ = 3.40 μM) were derived from the same isotherm profiles of the SIX3-GEMININ interaction. The K_D_ as determined by ITC was very similar to the value obtained from the SPR measurements.

**Fig. 2:**
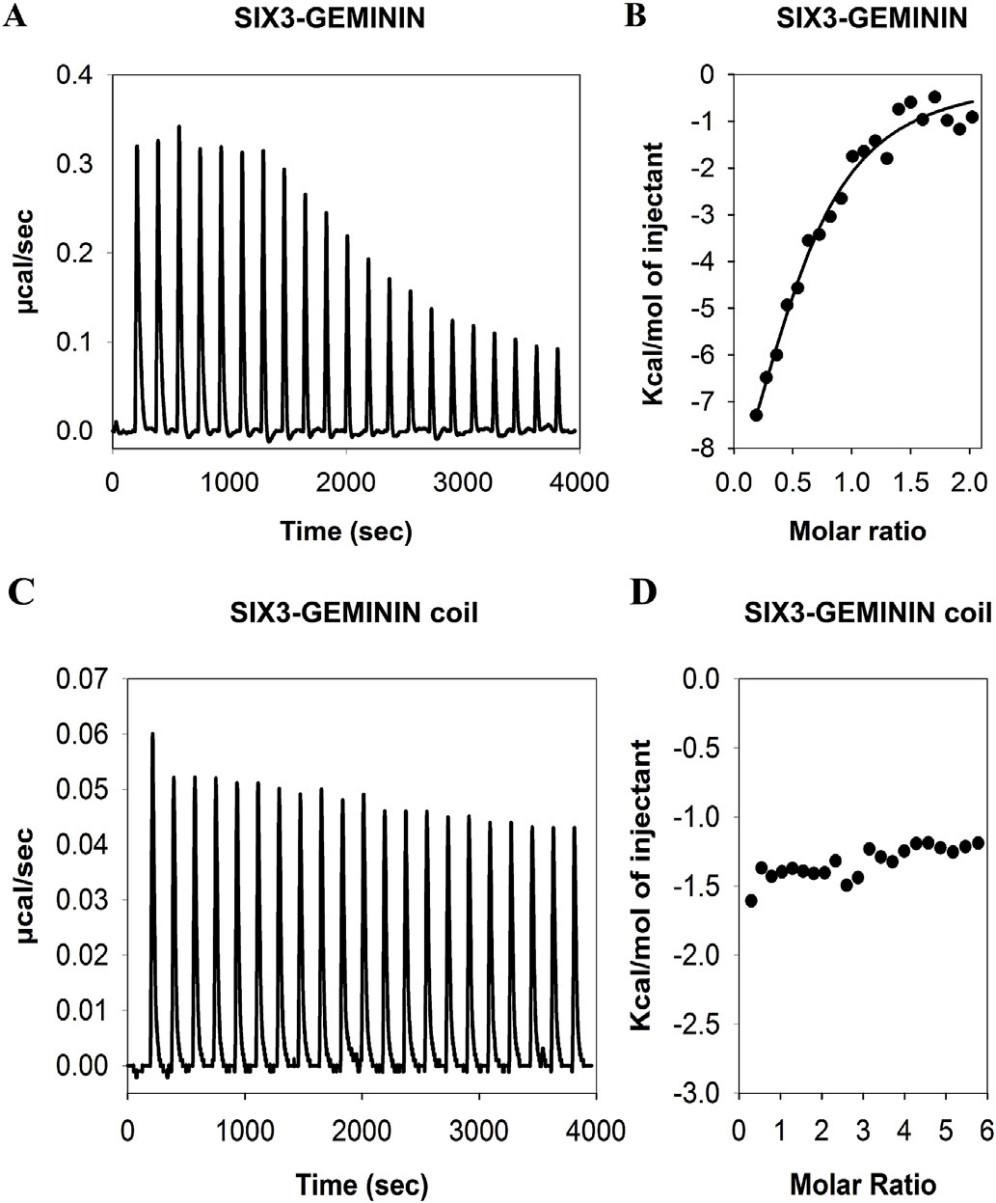
**ITC analysis of SIX3-GEMININ and SIX3-GEMININ coiled-coil**. A) Titration profiles of SIX3-GEMININ. Titration profiles were obtained by successive addition of GEMININ to MBPSIX3. B) Binding analysis SIX3-GEMININ. The area under each peak integrated and fitted to a one side Independent binding model of the SIX3-GEMININ after background (GEMININ to MBP-binding) subtraction: n = 0.55, K_D_ = 3.41 μM, ΔH = -317 kJ/mol, ΔS = -960 J/molK. C) Titration profiles of SIX3-GEMININ coiled-coil. Titration profiles were obtained by successive addition of the coiled-coil region of GEMININ to MBPSIX3. D) Binding analysis SIX3-GEMININ coiled-coil. No binding was observed.

The coiled-coil region of GEMININ, which functions as the protein-interacting domain for other GEMININ-interacting proteins such as CDT1 [16,17], did not interact with SIX3 under the same ITC experimental conditions (Fig. 2C and D). Consequently, this region was not included in SPR experiments. MBP-fused SIX3SD interacted poorly with GEMININ in ITC experiments and protein precipitate was found in the sample cell (data not shown).

### 3.3 Full-length SIX3/SIX6 proteins bind C- or N-terminal regions of GEMININ in living cells

The interactions of SIX3/SIX6 with GEMININ were further studied in mammalian (COS-1) cells using bimolecular fluorescence complementation (BiFC). First, we tested if full-length proteins of SIX3/SIX6 interact with full-length GEMININ by co-transfecting corresponding plasmids. Strong fluorescence signals were detected in cells, showing that full-length SIX3/SIX6 proteins bind full-length GEMININ in living cells. (Fig. S1, Fig. S2). Next, we tested which part(s) of GEMININ is responsible for the interaction by transfecting either *GEMININ C* (C-terminal region of GEMININ)*, GEMININ N* (N-terminal region of GEMININ) or *GEMININ coiled-coil* together with *SIX3/SIX6.* Clear BiFC signals were observed in the nucleus of the cells either with *GEMININ C* or *GEMININ N*, indicating that SIX3 - GEMININ C and SIX3 - GEMININ N, as well as SIX6 - GEMININ C and SIX6 - GEMININ N were formed (Fig. 3A, D, Fig. 4A, D). However, the cells transfected with *GEMININ coiled-coil* did not yield signals beyond the background level (Figs. 3G and 4G).

**Fig. 3:**
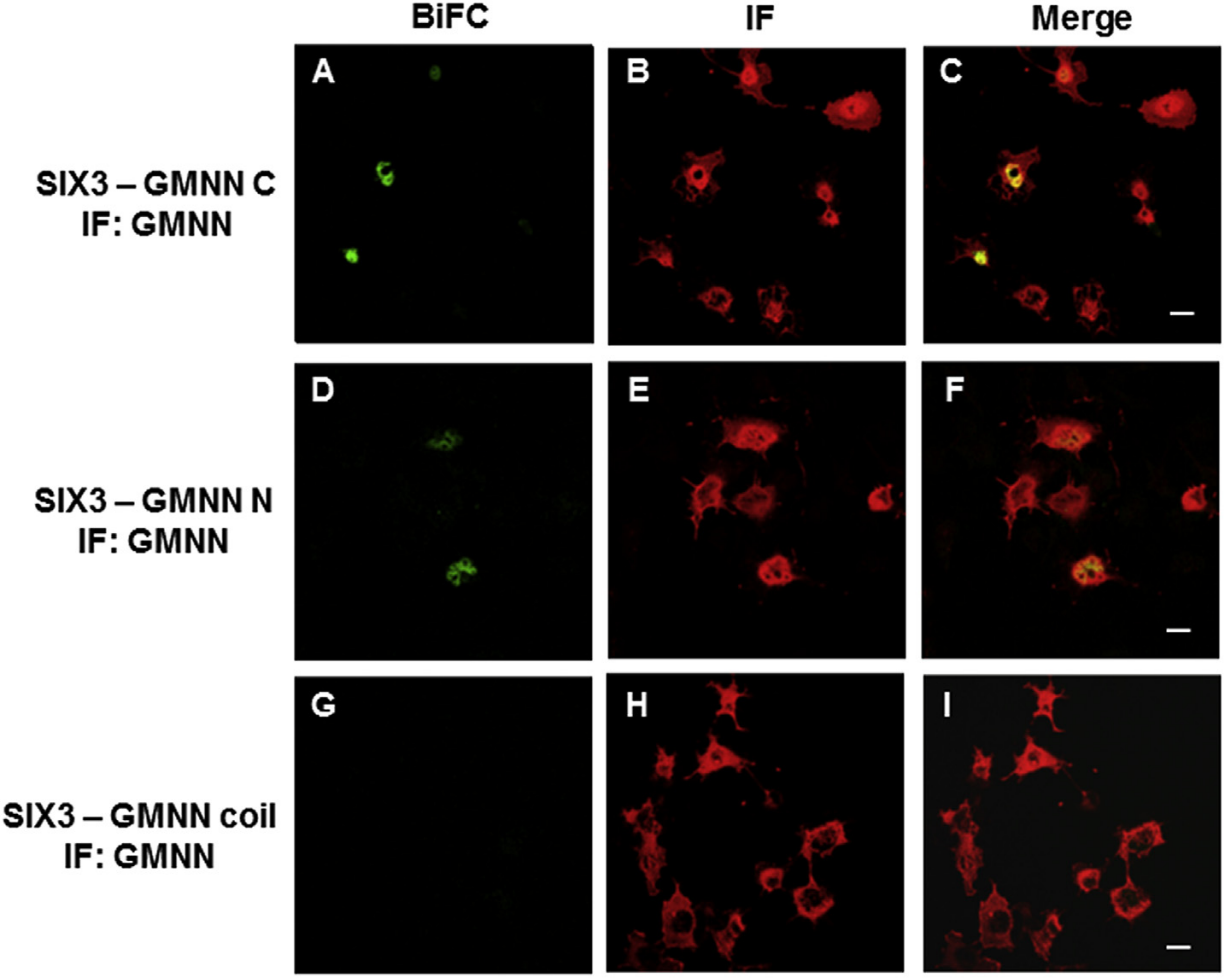
**GEMININ (GMNN) – SIX3 complex formation in living cells.** The interactions of the pBiFC-VC155 SIX3 to different pBiFC-VN155 (I152L) GEMININ constructs (C-terminal region of GEMININ, GMNN C; N-terminal region of GEMININ, GMNN N; coiled-coil domain of GEMININ, GMNN coil) were studied in co-transfected COS-1 cells. Shown are representative confocal images. A) BiFC image of the SIX3 – GMNN C complex, B) GMNN C immunostaining, D) BiFC image of the SIX3 – GMNN N complex, E) GMNN N immunostaining, G) BiFC image of the SIX3 – GMNN coil complex, H) GMNN coil immunostaining. C), F), I), Merged images. Scale bars, 20 μm.

**Fig. 4:**
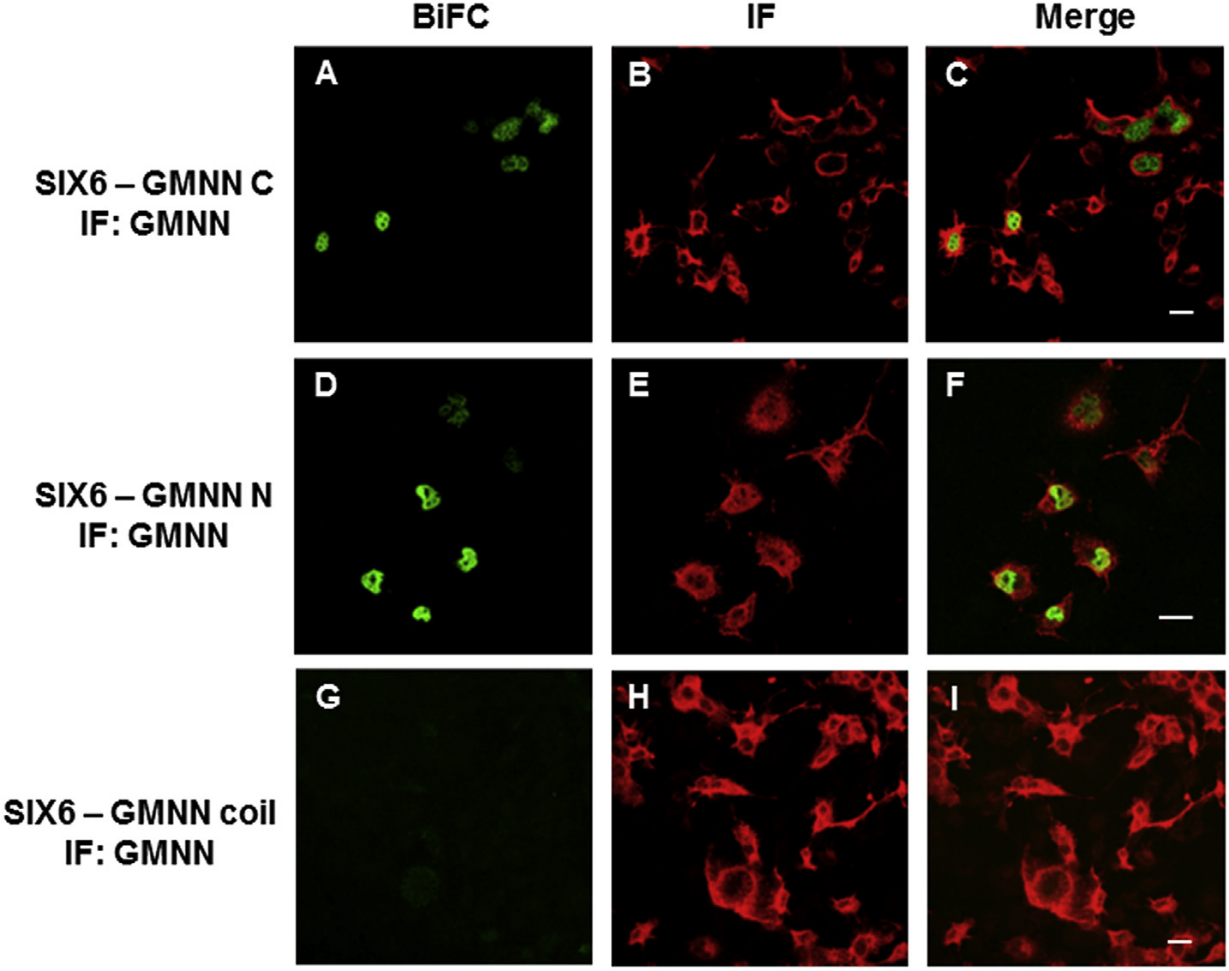
**GEMININ (GMNN) – SIX6 complex formation in living cells.** The interactions of the pBiFC-VC155 SIX6 to different pBiFC-VN155 (I152L) GEMININ constructs (C-terminal region of GEMININ, GMNN C; N-terminal region of GEMININ, GMNN N; coiled-coil domain of GEMININ, GMNN coil) were studied in co-transfected COS-1 cells. Shown are representative confocal images. A) BiFC image of the SIX6 – GMNN C complex, B) GMNN C immunostaining, D) BiFC image of the SIX6 – GMNN N complex, E) GMNN N immunostaining, G) BiFC image of the SIX6 – GMNN coil complex, H) GMNN coil immunostaining. C), F), I), Merged images. Scale bars, 20 μm.

**Fig. 5:**
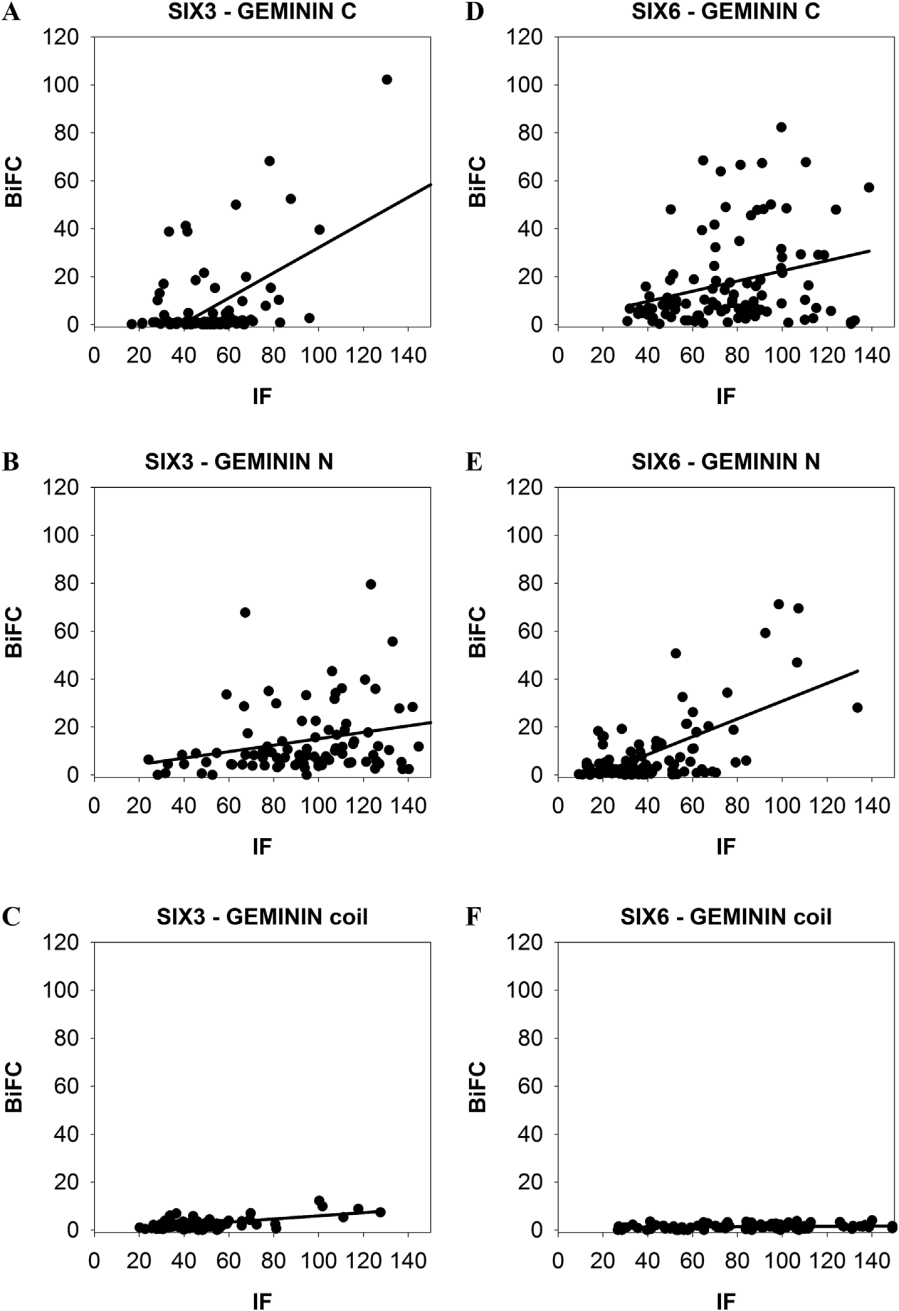
**Single-cell analysis of Geminin and SIX3/SIX6 -co-transfected COS-1 cells.** Mean pixel intensities for the GEMININ - SIX3/SIX6 BiFC signals are plotted against the immunofluorescence (IF) intensities for ~100 cells. A) SIX3 - GEMININ C (C-terminal region of GEMININ) complex formation. B) SIX3 - GEMININ N (N-terminal region of GEMININ) complex formation. C) SIX3 - GEMININ coil (coiled-coil domain of GEMININ) complex formation. D) SIX6 - GEMININ C complex formation. E) SIX6 - GEMININ N complex formation. F) SIX6-GEMININ coil complex formation.

The levels of these proteins were checked by staining the cells with specific antibodies. The immunostaining showed comparable levels of protein expression in each transfected cell population (Fig. 3B, E, F, Fig. 4B, E, F). To estimate the extent of the protein interaction, the BiFC signals (representing the complex formation) from single cells were plotted against the immunofluorescence signals (representing the protein expression levels) from the same cells (Fig. 5). BiFC signals varied between cell populations and were shown as the slopes of linear regression lines. For the C- and N- terminal constructs of GEMININ, a sharp slope was observed with m = 0.52 for GEMININ C - SIX3, 0.15 for GEMININ C - SIX6, 0.11 for GEMININ N - SIX3, 0.15 for GEMININ N - SIX6, (Fig. 5A, B, D, E), whereas the slope inclination was m = 0.02 for GEMININ coil - SIX3 and m = 0.00 for GEMININ coil - SIX6 (Fig. 5C, F). This indicates that GEMININ binds SIX3/SIX6 proteins via its C- and N- terminal regions.

### 3.4 The C-terminal region of SIX3/SIX6 proteins is required for its binding to GEMININ in living cells

**Fig. 6:**
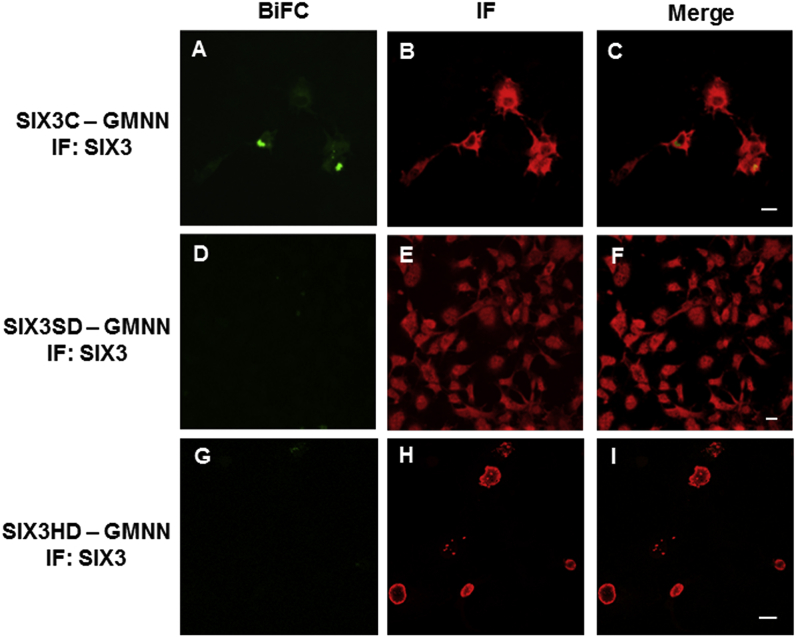
**SIX3 - GEMININ complex formation in living cells.** The interactions of the pBiFC-VC155 SIX3C (C-terminal region of SIX3), SIX3SD (SIX domain of SIX3) and SIX3HD (homeodomain of SIX3) to pBiFC-VN155 (I152L) GEMININ were studied in co-transfected COS-1  cells. Shown are representative confocal images. A) BiFC image of the SIX3C - GEMININ complex, B) SIX3C immunostaining, D) BiFC image of the SIX3SD - GEMININ complex, E) SIX3SD immunostaining, G) BiFC image of the SIX3HD - GEMININ complex, H) SIX3HD immunostaining. C), F), I), Merged images. Scale bars, 20 μm.

**Fig. 7:**
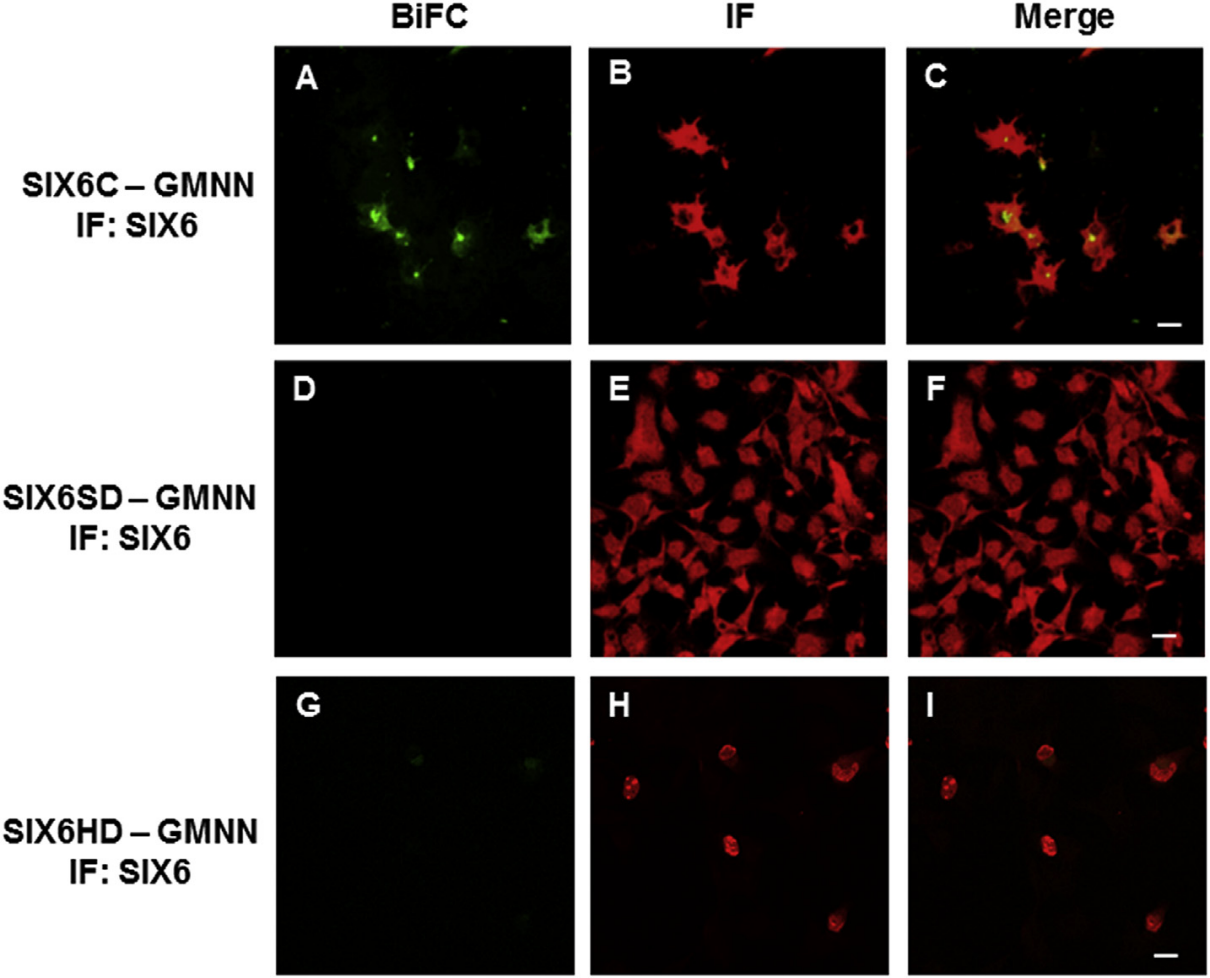
**SIX6 - GEMININ complex formation in living cells.** The interactions of the pBiFC-VC155 SIX6C (C-terminal region of SIX6), SIX6SD (SIX domain of SIX6) and SIX6HD (homeodomain of SIX6) to pBiFC-VN155 (I152L) GEMININ were studied in co-transfected COS-1 cells. Shown are representative confocal images. A) BiFC image of the SIX6C - GEMININ complex, B) SIX6C immunostaining, D) BiFC image of the SIX6SD - GEMININ complex, E) SIX6SD immunostaining, G) BiFC image of the SIX6HD - GEMININ complex, H) SIX6HD immunostaining. C), F), I), Merged images. Scale bars, 20 μm.

The SPR experiments showed that SIX3SD alone interacted with GEMININ much weakly than the full-length SIX3 did, (Fig. 1), therefore we further investigated which regions/domains of SIX3/SIX6 proteins (Fig. S3) may be responsible for the interactions in the living cells. BiFC signals were observed when the plasmids carrying either *SIX3C, (*C- terminal region of *SIX)3,* or *SIX6C, (*C-terminal region of *SIX6)* (Fig. 6, Fig. 7) were co-transfected with the *GEMININ-*carrying plasmids, indicating that both SIX3C - GEMININ and SIX6C - GEMININ complexes were formed (Figs. 6A and 7A). The interaction signals were detected both in the nucleus and in the cytoplasm. When the plasmids carrying *SIX3SD*/*SIX6SD* (SIX domain region of either *SIX3* or *SIX6*) or *SIX3HD/SIX6HD* (homeodomain region of either *SIX3* or *SIX6*) were used, fluorescent signals were not detected (Fig. 6D, G, Fig. 7D, G), indicating a lack of, or strongly reduced, interaction of the SIX domains or homeodomains alone with GEMININ in living cells. The antibody immunostaining showed similar levels of protein expression in each transfected cell population for the different *SIX3/SIX6* constructs tested (Fig. 6B, E, H, Fig. 7B, E, H). Their subcellular localisation patterns, however, were different; HDs were found in the nucleus alone, whereas both C-terminal regions and SDs were detected both in the cytoplasm and in the nucleus.

**Fig. 8:**
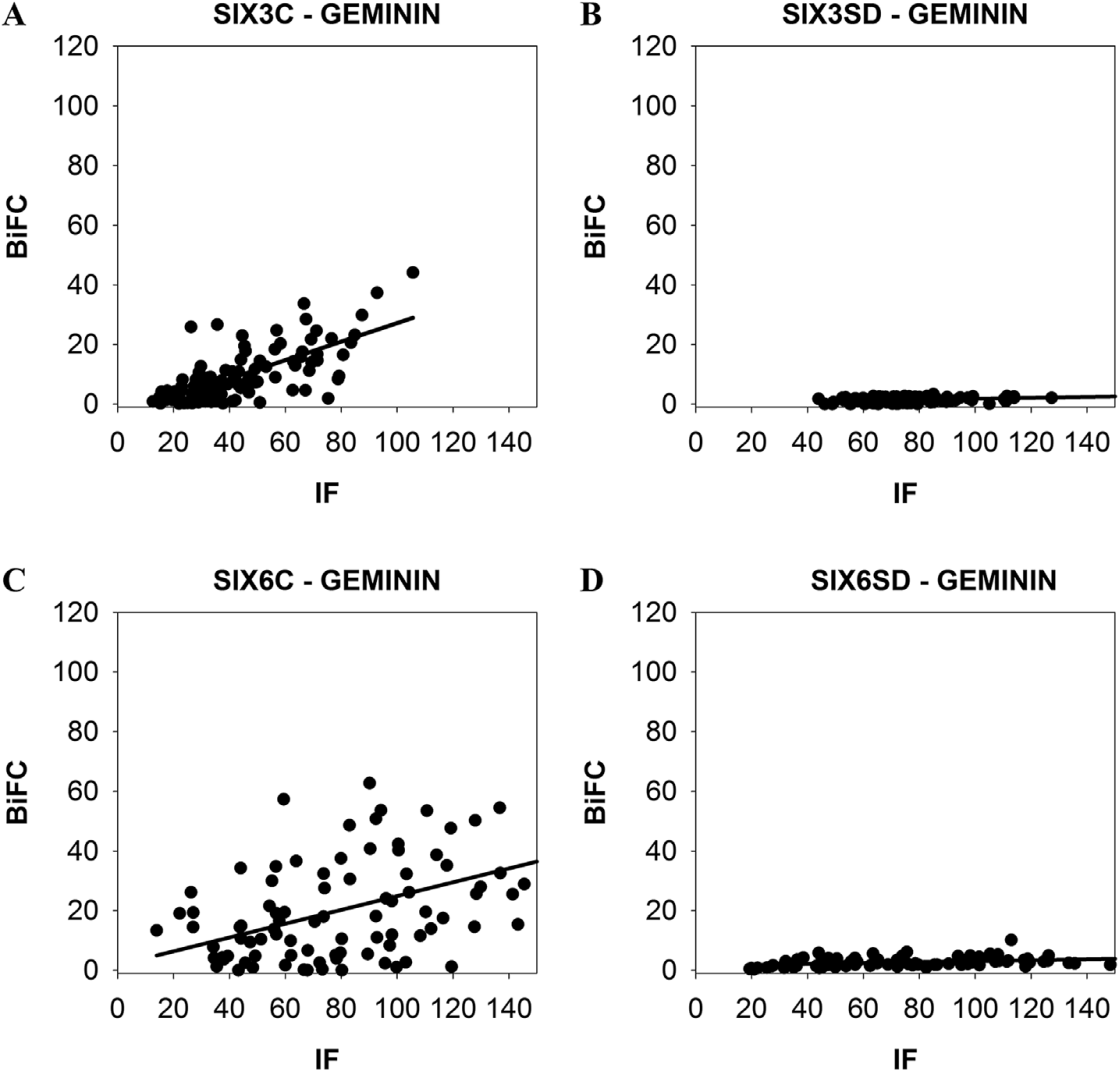
**Single-cell analysis of SIX3/SIX6 and Geminin-co-transfected COS-1 cells.** Mean pixel intensities for the SIX3/SIX6-GEMININ BiFC signals are plotted against the immunofluorescence (IF) intensities for ~100 cells. A) SIX3C (C-terminal region of SIX3) - GEMININ complex formation. B) SIX3SD (SIX domain of SIX3) - GEMININ complex formation. C) SIX6C (C-terminal region of SIX6) - GEMININ complex formation. D) SIX6SD (SIX domain of SIX6) - GEMININ complex formation.

The slopes of linear regression lines in the plots of the BiFC signals (representing the complex formation) from single cells against the immunofluorescence signals (representing the protein expression levels) from the same cells (Fig. 8) also clearly show the differences in the interactions. For the C-terminal regions of SIX3 and SIX6, the slope values were m = 0.31 for SIX3 and m = 0.23 for SIX6 (Fig. 8A, C), whereas the slope inclination was strongly reduced (m = 0.01) for SIX3SD and SIX6SD (Fig. 8B, D). This indicates that only the C-terminal regions of SIX3/SIX6 bind GEMININ specifically.

## 4 Discussion

The homeodomain-containing transcription factors SIX3 and SIX6 play important roles in eye and forebrain development. In understanding their roles in development, attention has been largely focused on the two evolutionary conserved domains: the homeodomain (DNA-binding domain) and the SIX domain (protein-protein interaction domain), although previous studies suggested possible functions in phosphorylation and DNA binding for the non-conserved C-terminal regions [15,28].

Using BiFC we investigated whether the interactions occur between GEMININ and SIX3/SIX6 testing homeodomains (HDs), SIX domains (SDs) or C-terminal regions of the SIX3/SIX6 proteins. We show here that SIX3/SIX6 interact directly with GEMININ through its C-terminal regions in living cells (Fig. 6, Fig. 7). The binding of GEMININ to the C- terminal regions of SIX3/SIX6 illustrates the significance of these unstructured regions in functional activities. The fact that SIX3SD did bind GEMININ considerably weaker (by SPR, Fig. 1) and SIX3HD did not bind GEMININ-HBR (by ITC) [21], indicates that the C-terminal region of SIX3 contributes most, if not all, to the interaction with GEMININ. Therefore, both *in vitro* and *in cellula* protein binding studies support the notion that the SIX domain alone is not sufficient or may not even be required for SIX3/6-GEMININ interaction. The functional importance of the C-terminal region was also implied from the mutational analysis of *SIX3*, a major causative gene in holoprosencephaly. Namely, mutations both in structured domains of SDs and HDs as well as in the unstructured C-terminal region of the SIX3 protein resulted in phenotypic abnormalities [29,30].

The involvement of the unstructured C-terminal regions of a SIX protein in protein-protein interaction has not been reported until the present study. The SIX1 protein, a homologue of SIX3/SIX6, binds to the EYA2 protein through the α1 helix of the SIX domain [14]. The involvement of the SIX domain in the SIX1-EYA2 interaction was expected since it has been generally accepted as a protein-interacting domain of the SIX proteins. Because the SIX1 construct used in the SIX1-EYA2 study contained only the SIX domain and the homeodomain, but not the C-terminal region, the involvement of the C-terminal region of a SIX protein in protein interaction was not tested. SIX3 forms a complex with LSD1, a histone modifying enzyme, and MAT3, a metastasis-associated family protein which is a part of the nucleosome remodeling and deacetylation (NuRD) complex [31]. The GST-pulldown assays showed that MTA3 binds to the N-terminal region and the SIX domain of SIX3, and LSD1 binds to the homeodomain including the last 25 residues of the SIX domain. However, no bindings occurred when these SIX3-interacting partners were tested with the C-terminal region of SIX3.

Fluorescence microscopy revealed that the different regions of SIX3/SIX6 proteins displayed discrete subcellular distributions (Fig. 6, Fig. 7). The HDs were mainly located in the nucleus, whereas both the C-terminal regions and the SDs were detected in both the nucleus and the cytoplasm. Our data suggests that a nuclear import-export shuttling mechanism for SIX3 and SIX6 proteins between the cytoplasm and the nucleus may exist and temporal and spatial specificity may involve some other features, likely phosphorylation at the C-terminal regions of SIX3/SIX6. For SIX1, another member of the SIX class protein, the cell-cycle regulated phosphorylation by casein kinase II (CK2), a serine/threonine kinase, is indeed the case [28]. Since SIX3/SIX6 also have putative phosphorylation sites, the same shuffling mechanism may be in operation. Supporting this, the interaction of Geminin with Hox and the resulted inhibition of Hox transcriptional activities are also modulated by cell-cycle dependent nuclear-cytoplasmic shuttling [32] through phosphorylation of residue S184 in the C terminus of Geminin by CK2 [21].

Similar binding affinities of SIX3 to GEMININ were obtained using the SPR and ITC techniques, supporting the validity of these *in vitro* measurements. Moreover, the very negative entropy (-956 J/molK) and high enthalpy (-317 kJ/mol) suggests a disordered to ordered transition upon binding [33,34]. The rather low binding affinity (~ 3 μM) also supports an induced folding mechanism where the coupled binding and folding determines a high specificity and low affinity combination [35]. The lack of interaction between SIX3 and the well-known protein-binding coiled-coil region of GEMININ was supported by both ITC and BiFC (Fig. 2C and D; Fig. 3G, I). (Rather the N- and C-terminal regions of GEMININ interacted with the SIX3/SIX6 protein (Fig. 3A, D, Fig. 4A, D). Interestingly, CDT1, a cell-cycle determinant and a competitor of SIX3 as one of major interacting partners of GEMININ, does bind to the coiled-coil region of GEMININ [5,16,19]. Since the binding stoichiometry of 1:2 for GEMININ was the same for both SIX3 and CDT1 (i.e., 1 molecule of SIX3 or CDT1 versus 2 chains of GEMININ), it appears that CDT1 and SIX3 bind different regions of GEMININ, yet prevents each other from binding to GEMININ. An allosteric modulation could explain this, but this possibility has not been tested. Also the fact that the intrinsically disordered regions often have many interaction partners [36], other interacting partners of SIX3/SIX6 may also bind to their C-terminal regions.

To characterise the SIX3-GEMININ interaction further, it is necessary to pinpoint the interacting residues of both proteins. An indirect method such as NMR would be a good choice, as NMR will also reveal a possible unstructured to structured transition. However, as the recombinant SIX3 and SIX6 proteins are highly unstable and require high salt buffers to avoid aggregation, keeping them stable and soluble remains a challenge. To increase solubility and avoid aggregation, one would have to use high-salt buffer (minimum 400 mM NaCl), but the high salt also strongly reduces NMR signals.

In conclusion, we have shown that the C-terminal regions of SIX3 and SIX6, not the SIX domain or homeodomain alone, bind GEMININ and the binding involves the N- or C-terminal region of GEMININ. The coiled-coil region, which is a well-established protein interaction domain present in GEMININ, is not involved in the binding.

## Figures and Tables

**Fig. 1:**
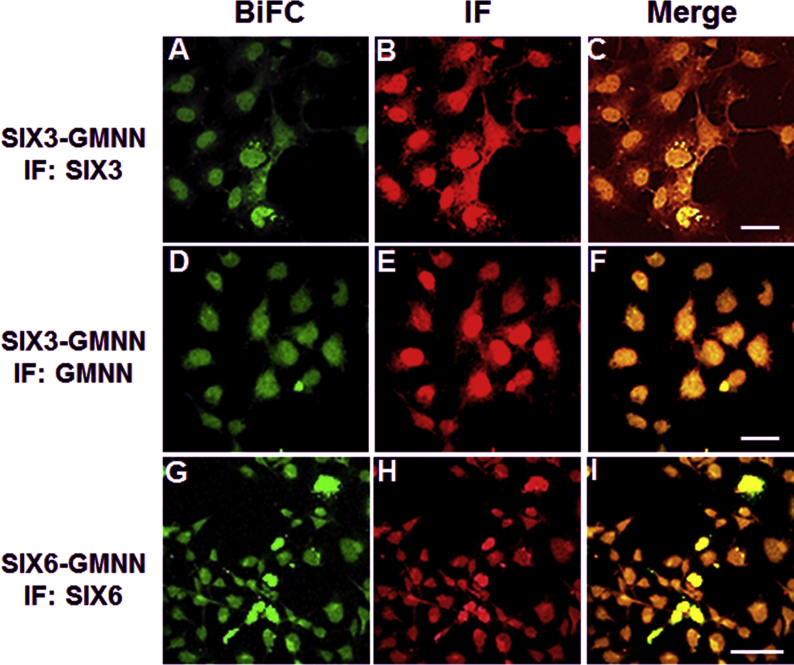
**SIX3/SIX6 - GEMININ complex formation in living cells.** The interactions of the pBiFC-VC155 SIX3 and SIX6 to pBiFC-VN155 (I152L) GEMININ were studied using BiFC in co-transfected COS-1 cells. Shown are representative confocal images. A) BiFC image of the SIX3-GEMININ complex, B) SIX3 immunostaining, D) BiFC image of the SIX3-GEMININ complex, E) GEMININ immunostaining, G) BiFC image of the SIX6-GEMININ complex, H) SIX6 immunostaining. C), F), I) Merged images. Scale bars, 15 μm

**Fig. 2:**
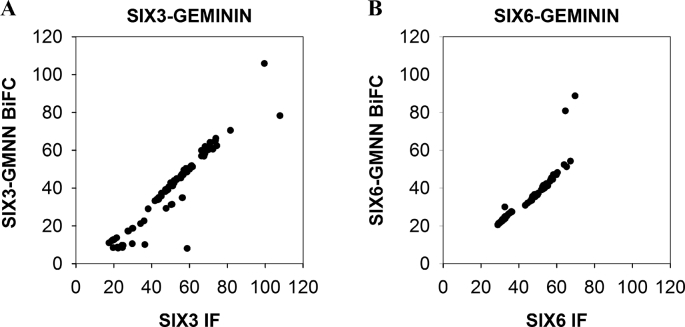
**Single-cell analysis of SIX3/SIX6 and Geminin-cotransfected COS-1 cells**. Mean pixel intensities for the SIX3/SIX6-GEMININ BiFC signals are plotted against the SIX3/SIX6 immunofluorescence (IF) intensities for ~100 cells. A) SIX3-GEMININ complex formation. B) SIX6-GEMININ complex formation

**Fig. 3:**
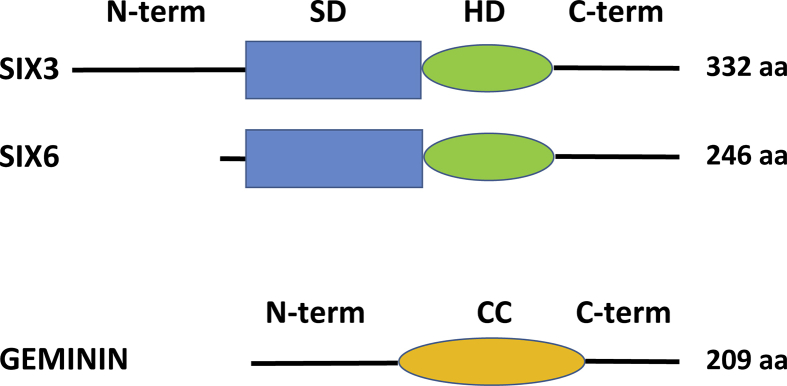
**Schematic representation of the structure of the SIX3/SIX6 and GEMININ proteins.** Structural elements of the SIX3/SIX6 include a well‐conserved Six domain (SD) and a homeodomain (HD) that is responsible for DNA binding. Structural elements of GEMININ include the coiled-coil domain (CC).

**Fig. 4:**
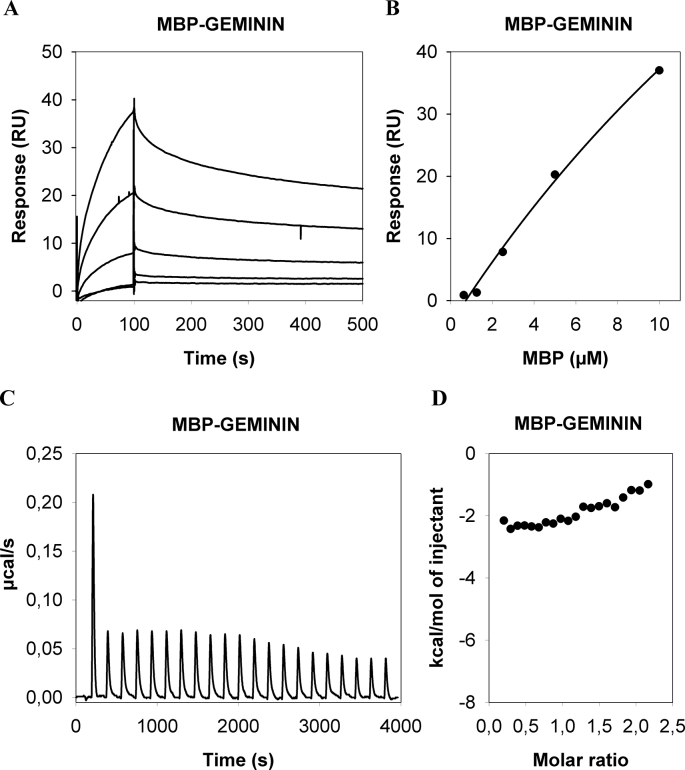
**SPR and ITC analysis of MBP with GEMININ.** A) MBP-GEMININ Sensorgram. MBP, at varying concentrations of 0.62 μM, 1.25 μM, 2.5 μM, 5 μM, 10 μM, was injected into the GEMININ-containing flow cell and the reference flow cell for 100 s. B) MBP-GEMININ binding affinity curve: K_D_: 35.3 μM, R_max_: 185.3, Chi^2^ (RU^2^): 2.6. C) ITC titration profiles of MBP-GEMININ. Titration profiles were obtained by successive addition of GEMININ to MBP. B) Binding analysis MBP-GEMININ. No binding was observed.
